# What haplodiploids can teach us about hybridization and speciation

**DOI:** 10.1111/mec.13393

**Published:** 2015-10-18

**Authors:** Konrad Lohse, Laura Ross

**Affiliations:** School of Biological Sciences, Institute of Evolutionary Biology, University of EdinburghEdinburgh, EH9 3JT, UK

**Keywords:** comparative biology, hybridization, insects, population genetics -theoretical biology, speciation

## Abstract

Most evolutionary theory focuses on species that reproduce through sexual reproduction where both sexes have a diploid chromosome count. Yet a substantial proportion of multicellular species display complex life cycles, with both haploid and diploid life stages. A classic example is haplodiploidy, where females develop from fertilized eggs and are diploid, while males develop from unfertilized eggs and are haploid. Although haplodiploids make up about 15% of all animals (de la Filia *et al*. 2015), this type of reproduction is rarely considered in evolutionary theory. In this issue of Molecular Ecology, Patten *et al*. (2015) develop a theoretical model to compare the rate of nuclear and mitochondrial introgression in haplodiploid and diploid species. They show that when two haplodiploid species hybridize, nuclear genes are much less likely to cross the species barrier than if both species were to be diploids. The reason for this is that only half of the offspring resulting from matings between haplodiploid species are true hybrids: sons from such mating only inherit their mother genes and therefore only contain genes of the maternal species. Truly, hybrid males can only occur through backcrossing of a hybrid female to a male of one of the parental species. While this twist of haplodiploid transmission genetics limits nuclear introgression, mitochondrial genes, which are maternally inherited, are unaffected by the scarcity of hybrid males. In other words, the rate of mitochondrial introgression is the same for haplodiploid and diploid species. As a result, haplodiploid species on average show a bias of mitochondrial compared to nuclear introgression.

Patten *et al*.'s (2015) study impressively demonstrates the explanatory power and potential for surprise of theoretical work. Although their model is beautifully simple, it contains all the key ingredients that have previously been considered to affect the ratio of mitochondrial to nuclear introgression in diploid taxa: Males and females may differ in their dispersal ability, and hence the potential to generate hybrids in the first place. More importantly, hybrid males and females may differ either in fitness due to intrinsic incompatibilities (Haldane's rule) or in their ability to backcross to the parental species (due to prezygotic barriers). Surprisingly, however, Patten *et al*. (2015) find that in haplodiploids, most of these details are irrelevant and that nuclear introgression is reduced relative to mitochondrial introgression (and nuclear introgression in diploid taxa) over most of parameter space. In particular, nuclear introgression is reduced even when Haldane's rule does not hold, that is, when male hybrids have equal or even higher fitness than female hybrids. The only way to avoid the reduction in nuclear introgression is if there is a strong male bias in the migrant pool or the backcross probability for hybrid females is much smaller than that of hybrid males.

The results of Patten *et al*. (2015) are not only surprising, but also help make sense of several empirical studies that have found striking incongruencies between nuclear and mitochondrial gene trees in haplodiploid taxa (Rokas *et al*. 2003; Linnen & Farrell 2007; Nicholls *et al*. 2012; Wachi *et al*. 2012). However, attributing such incongruencies to mitochondrial-biased introgression is challenging for at least two reasons; (i) both incomplete lineage sorting and introgression can lead to incongruent gene trees, (ii) mitochondria are inherited as a single linked locus which has a highly random genealogy. Therefore, inference methods based on the coalescent which explicitly model the randomness of the genealogies for histories involving introgression are required to test for differences in the rate of introgression between mitochondrial and autosomal genes. For example, Linnen & Farrell 2007 used IM (Hey & Nielsen 2004) to separately estimate the rate of introgression for mitochondrial and nuclear genes in a group of *Neodiprion* pine sawflies and found a strongly increased rate of mitochondrial introgression (Fig.1a). The same has been found in *Andricus* oak gall wasps (Fig.1b) (Wachi *et al*. 2012). While these results are tantalizing in the light of Patten *et al*.'s findings, there are of course other factors, in particular female-biased dispersal and sweeps induced by endosymbionts such as *Wolbachia* (Hurst & Jiggins 2005) that can lead to incongruencies between nuclear and mitochondrial genealogies and make individual case studies hard to interpret. Unfortunately, there is little data from haplodiploid clades outside the Hymenoptera: a study on two species of haplodiploid spider mites found that they where polyphyletic for a mitochondrial marker, yet monophyletic for a nuclear marker (Navajas & Boursot 2003), providing support for Patten's findings, while on the other hand, no mitochondrial incongruence was observed in another haplodiploid clade, the armoured scale insect (Hemiptera: Diaspidae) (Gwiazdowski *et al*. 2011).

**Figure 1:**
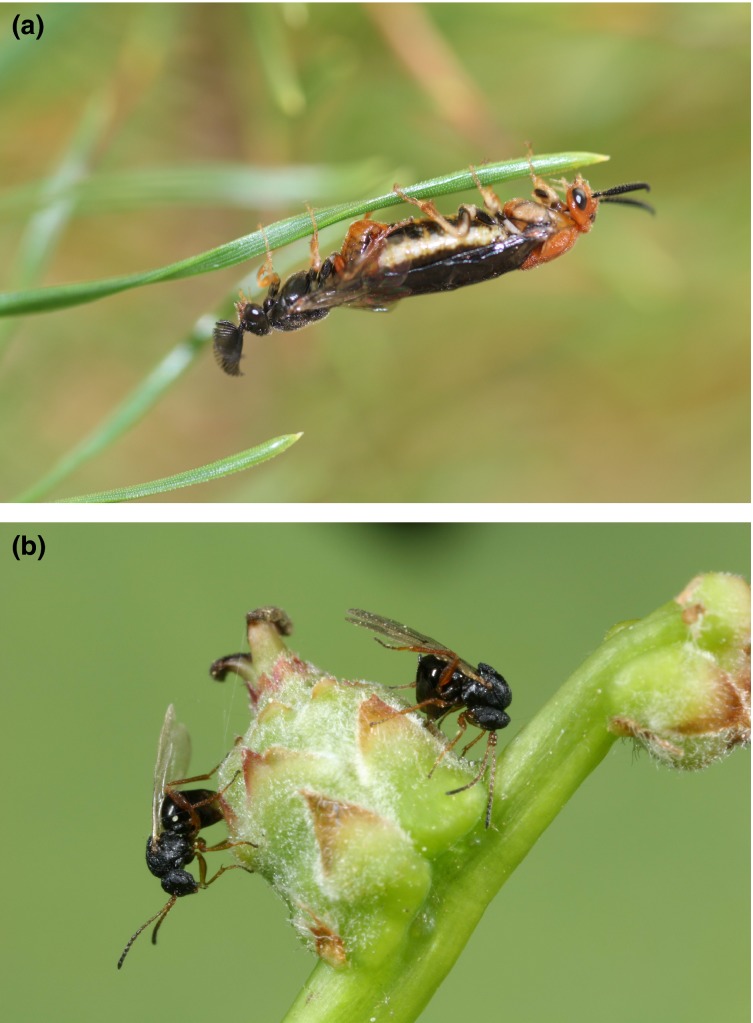
Mitochondrial introgression far exceeds nuclear introgression in hybridizing pairs of (a) *Neodiprion* species (pictured here is a mating pair of *N. lecontei,* photograph by Robin Bagley) and (b) *Andricus* oak gall wasps (photograph by György Csóka).

The expected bias towards mitochondrial introgression in haplodiploid taxa also has implications for the interpretation of mitochondrial sequence data in phylogeographic and DNA barcoding studies in haplodiploid taxa. Patten *et al*. (2015) show that, the argument that mitochondrial barcodes should more closely reflect the history of species and populations than nuclear gene trees simply may not apply in hybridising haplodiploid taxa and one expects to see more incongruence between mitochondrial and nuclear gene trees in such cases than in diploid taxa. More phylogenetic studies that explicitly test for incongruences between nuclear and mitochondrial markers are needed to test whether this is indeed the case.

Patten *et al*.'s model may also be relevant for the evolution of X-chromosomes in diploid organisms, which have the same transmission pattern as nuclear genes in haplodiploids. Simple extrapolation of the results would predict lower levels of X-chromosome introgression compared to autosomes. This would fit well with empirical patterns observed in a wide range of species that are generally attributed to other factors such as Haldane's rule, recessive alleles or faster-X (Presgraves 2008). However, this analogy is too simplistic: under haplodiploidy, male offspring of interspecific matings only contain maternal genes, while F1 diploid males contain the maternal genotype only at their X-chromosome and so are affected by X-autosome incompatibilities or may differ in mating preference from males of the parental species. So although the haploid transmission genetics of the X could be a tantalizing alternative explanation for low X-linked introgression, more formal theory is clearly necessary to explore this possibility.

Perhaps the most fascinating upshot of Patten *et al*.'s work is that it may explain the abundance of haplodiploid species. If haplodiploidy by itself stymies nuclear introgression, it may be easier to generate and maintain new species under haplodiploidy than diploidy. Testing this will require making use of haplodiploid groups outside the Hymenoptera. There are more than 20 independent origins of haplodiploidy among invertebrates, in principle allowing phylogenetically controlled comparisons between haplodiploid and diplodiploid clades. For example, comparing the number of species in reciprocally monophyletic diploid and haplodiploid sister clades of insects shows that haplodiploidy is indeed more often (in five vs. two cases) associated with greater species diversity (Table1) as expected by Patten *et al.’*s model [note that we excluded all interorder comparisons such as between Hymenoptera and its sister clade]. However, the ongoing explosion of sequence data and rapid development of statistical tools to reconstruct past speciation histories (see Sousa & Hey 2013; for a review) from such data, hold the promise of exploring the consequences haplodiploidy has for the speciation process much more directly. For example, one can envisage systematic comparisons of the magnitude of postdivergence gene flow between haplodiploid and diploid taxa. Combining these with laboratory-based measurements of hybrid fitness and using the model of Patten *et al*. (2015) would give a way to estimate the strength of sex-specific prezygotic barriers. It seems that we can learn a great deal about introgression and speciation in general by paying closer attention to haplodiploid taxa.

**Table 1:** Species number comparison between haplodiploid and pseudohaplodiploid (PGE) clades and their diploid sister groups for each independent origin of haplodiploidy of among insects. Rows in bold represent within order comparisons

Order/Class	Haplodiploid clade	Type of haplodiploidy*	Species number	Sister group	Species number	Haplodiploid clade more species?
**Coleoptera**	**Micromalthus**	**Arrhenotoky**	**1**	**Cupedidae**	**30**	**−**
**Coleoptera**	**Xyleborini**	**Arrhenotoky**	**1360**	**Coccotrypes**	**120**	**+**
**Coleoptera**	**Hypothenemus**	**PGE**	**179**	**Allernoporus**	**1**	**+**
Collembola	Symphypleona	PGE	1188	Neelipleona	33	+
**Diptera**	**Sciaridae+Cecidomyiidae**	**PGE**	**8,468**	**Keroplatidae**	**945**	**+**
**Hemiptera**	**Aleyrodidae**	**Arrhenotoky**	**1550**	**Aphididae+Coccoidea**	**12400 (5400)**†	**− (−)**
**Hemiptera**	**Iceryini**	**Arrhenotoky**	**81**	**Gueriniella**	**2**	**+**
**Hemiptera**	**Neococcoidea**	**PGE**	**7000**	**Putoidae**	**50**	**+**
Hymenoptera	Hymenoptera	Arrhenotoky	115,000	Other Holometabola	735000	**−**
Phthiraptera‡	Phthiraptera	PGE	3000	Psocoptera	5500	**−**
Thysanoptera	Thysanoptera	Arrhenotoky	5000	Hemiptera	50000 (41369)†	**−** (**−**)

* Arrhenotoky: females develop from fertilized eggs and are diploid, while males develop from unfertilized eggs and are haploid. PGE: Paternal genome elimination or pseudohaplodiploidy, where both sexes develop from fertilized eggs, but where paternal origin genes are eliminated, either from just the germline resulting in diploid males, or from both soma and germline resulting in haploid males.

† The sister group contains haplodiploid species that were either included or excluded (within brackets) from the comparison.

‡ PGE per se has only been described in a single species, but data on 14 other species show the unusual type of spermatogenesis that might be indicative of PGE.
